# How to develop viruses into anticancer weapons

**DOI:** 10.1371/journal.ppat.1006190

**Published:** 2017-03-16

**Authors:** Roberto Cattaneo, Stephen J. Russell

**Affiliations:** Department of Molecular Medicine and Virology and Gene Therapy Graduate School track, Mayo Clinic, Rochester, Minnesota, United States of America; University of Florida, UNITED STATES

## Introduction

Viruses have shaped human history through devastating infections. In addition, virus infection may be responsible for up to 15% of cancer deaths [1]. Nevertheless, certain viruses can be our “friends.” At the end of the 18th century, Edward Jenner used cowpox to protect humans against infection with a lethal pathogen, smallpox. Based on the effectiveness of this “vaccination” process, in the 1960s, the World Health Organization mounted a global vaccination campaign that resulted in the eradication of smallpox [2].

In the mid-20th century, the principle of virus attenuation through adaptation to unnatural hosts was extended to cultured cells: cells from different species were used to select viruses with multiple mutations, reducing replication speed and allowing the immune system to control viral infection. Based on such a “live-attenuated” vaccine, global eradication of another viral disease, rinderpest, was recently achieved [3]. Other global vaccination campaigns, including those against polio and measles, are progressing. In addition, subunit vaccines are proving to be effective against virus-induced cancers, preventing hepatitis B virus–induced hepatocellular carcinoma and human papilloma virus–induced cervical cancer [2, 4].

A new frontier is to develop viruses into anticancer weapons. Many cancers remain incurable despite recent advances in radio-, chemo-, and immunotherapy. Based on their preferential replication in tumor cells, viruses from nine families have progressed to clinical trials of oncolysis: DNA viruses include *Adenoviridae*, *Herpesviridae*, *Parvoviridae*, and *Poxviridae* and RNA viruses *Paramyxoviridae*, *Picornaviridae*, *Reoviridae*, *Retroviridae*, and *Rhabdoviridae* [5, 6]. Recently, a genetically modified herpes simplex virus 1–based oncolytic vector, named talimogene laherparepvec (T-VEC), was approved as cancer therapeutic in the United States and Europe [7]. What are the mechanisms supporting cancer therapy with viruses, and how can oncolytic virotherapy be improved?

## What makes a good oncolytic virus platform?

Shortly after the discovery of animal viruses, observing physicians reported cancer regressions coincident with natural infections, most notably in patients with lymphomas and leukemias who were suffering from viral hepatitis, glandular fever, chickenpox, or measles. Intentional transmission of virus infections was then pursued in a range of cancer types using several different virus isolates (most notably West Nile, mumps, and adenovirus) and led to definite tumor regressions but sometimes also to fatal encephalitis, as with West Nile virus in immunosuppressed lymphoma patients [8]. When tissue culture systems for animal cells were established, it became clear that many viruses grow much better in cancer cell lines than in primary cells. In retrospect, the multistep process of tumor pathogenesis [9] accounts for preferential virus spread by weakening multiple cellular responses to viral infections.

Every oncolytic virotherapy platform has advantages and disadvantages, and time will tell which one is most successful for each cancer type. Clearly, a promising approach to oncolysis is to match different viruses with tumor types naturally permissive for their replication. On the other hand, the broad natural receptor tropism of some platforms (for example, vaccinia- and vesicular stomatitis virus–based vectors) allows application towards many different types of cancer, an important consideration because of the large investments needed to generate and validate any new platform. This implies that it is best to initially develop a broadly applicable platform and then to target it. For example, the vaccine lineage–based measles virus (MeV) platform we have developed can enter many cell types through the ubiquitously expressed protein CD46, while future clinical trials may be based on viruses with targeted tropism.

The MeV platform stands on four premises. First, natural oncolytic properties: independent clinical observations documented cancer regression following wild-type measles infections, and oncolytic efficacy was then demonstrated in several animal cancer models [10]. Second, targeted cell entry and cell–cell fusion: the two-protein MeV membrane fusion system, which naturally operates with different receptors [11], can be modified for targeted entry into cancer cells, leading to cell–cell fusion and killing of neighboring cells. Third, pre- and postentry targeting options like particle activation through cancer-specific proteases and cancer-selective replication through modified interactions with host innate immunity proteins and microRNAs [5]. Fourth, safety: live-attenuated MeV has been administered as a vaccine to at least 1,000,000,000 children with outstanding safety and efficacy records [12].

## What has been achieved in the clinic?

Reasons for success or failure of early clinical trials with oncolytic viruses were difficult to assess because viral replication could not be easily monitored in humans. Many second-generation oncolytic viruses express reporter proteins that allow noninvasive monitoring of viral infection. For example, one oncolytic MeV (MV-CEA) expresses the soluble carcinoembryonic antigen (CEA) that is secreted in the blood stream, providing for noninvasive monitoring of the total amount of viral replication in the body. Another virus (MV-NIS) expresses the human thyroidal natrium iodine symporter (NIS), the physiological function of which is to transport iodide ions into cells. When NIS is expressed from the genome of an oncolytic virus, infected cells concentrate iodide or similar isotopes. Thus, NIS expression, which has been exploited for decades in clinical practice for thyroid imaging and ablation, can provide anatomical information about the location of virus-infected cells. Production of clinical grade MV-CEA and MV-NIS stocks was nontrivial because cancer trials operate with the equivalent of up to 10^8^ vaccine doses (10^11^ infectious units) per individual (Table 1) [7].

**Table 1:** Clinical trials with oncolytic measles viruses.

*Type of cancer / NCT number*	*Virus used (dose level)*	*Patients treated*	*Median OS (range)*
Ovarian / 00408590	MV-CEA (10^3^–10^7^ TCID_50_)	15	10.6 (1.3–79.9)
	MV-CEA (10^8^–10^9^ TCID_50_)	6	38.4 (7.2+–83.5+)
	MV-NIS (10^8^–10^9^ TCID_50_)	16	26.5 (7.0–44.4+)
Ovarian / 02364713	MV-NIS (10^9^ TCID_50_)	Recruiting (randomized phase II trial)	
Multiple myeloma / 00450814	MV-NIS (10^6^–10^11^ TCID_50_)	45	ongoing
Glioblastoma / 00390299	MV-CEA (10^5^–10^7^ TCID_50_)	20	ongoing
Mesothelioma / 01503177	MV-NIS (10^8^–9 x 10^9^ TCID_50_)	12	ongoing
Head and neck / 01846091	MV-NIS (10^8^ TCID_50_)	9	ongoing

*p* = 0.047 for OS superiority in high dose (10^8^–10^9^) versus low dose (10^3^–10^7^ cohorts).

NCT, ClinicalTrial.gov registry number; OS, overall survival, in months.

Both vectored MeV were engineered in the vaccine lineage genetic backbone used to establish reverse genetics [13]. Since they can enter cells and spread through the ubiquitous regulator of complement activation CD46, which is overexpressed in cancer cells, they are used in clinical trials against different cancer types, including ovarian cancer [14], multiple myeloma, glioblastoma multiforme, mesothelioma, and head and neck squamous cellular carcinoma (Table 1). Even if phase I and II clinical trials assess safety rather than efficacy, the data indicate that MeV-based cancer treatment is associated with increased median overall survival in patients with ovarian cancer and that survival was longest in the patient groups treated with the most virus [14]. Of major significance in these ovarian cancer patients, there was clear evidence for amplification of T cell responses against known ovarian tumor antigens after intraperitoneal MV-NIS administration, even though all patients were measles-immune pretherapy.

Perhaps the most striking hint of clinical efficacy was the durable complete remission at all disease sites documented in a patient with multiple myeloma after systemic treatment with 10^11^ MeV infectious units (100 ml of a 10^9^ infectious units/ml solution); this patient remains in remission more than three years after treatment [15]. Importantly, this and another patient who initially responded well had very low pretreatment measles antibody titers less than or equal to ten. For natural measles infection, a titer of measles antibody of 1,052 is considered fully protective, less than 120 is considered nonprotective, and between 120 to 1,052 is considered partially protective. These clinical observations have confirmed that widespread antimeasles immunity, while adding safety for the patient and contacts, can interfere with the efficacy of a systemically administered oncolytic virus.

## How to circumvent antiviral immunity?

Efficacy of oncolysis can be enhanced by pharmacological down-modulation of preexisting and induced antiviral immune responses, as demonstrated preclinically [5]. Nevertheless, more incisive solutions to overcome the neutralization barrier are sought. These include virus delivery through carrier cells and replacement or resurfacing of viral capsids or envelopes so that preexisting neutralizing antibodies are not effective.

Following infection, there is an eclipse period before the viral glycoproteins appear on the surface of an infected cell or viral capsids are released. Hence, infected cells can be used as virus delivery vehicles [16]. Virus-infected mesenchymal stem cells (MSC), cytokine-induced killer cells, monocytes, dendritic cells, and irradiated tumor cells have each been shown to be capable of delivering efficacious oncolytic virus infections to sites of tumor growth in mice that have been passively immunized with antiserum sufficient to negate the efficacy of oncolytic virus treatment delivered as particles. Indeed, a phase I clinical trial is underway in ovarian cancer patients testing repeated intraperitoneal administration of autologous MV-NIS–infected adipose-derived MSC (ClinicalTrials.gov identifier: NCT 02068794).

For many viruses, an effective method of escaping neutralizing antibodies is envelope exchange, whereby a different (typically less than 60% homologous) serotype of the glycoproteins is used in place of the original one [5]. While these successes may offer a solution to the problem of high virus seroprevalence, they also raise concerns regarding the safety of using a virus chimera in clinical trials.

## How to improve efficacy of oncolytic viruses while maintaining safety?

With some exceptions, cancer therapies based on replicating viruses have been tolerated well after both local and systemic administration [6]. No virus transmission from treated patients to medical personnel or other contacts has been reported, although viral RNA sequences (but not infectious virus) have been detected in saliva and urine, especially after high-dosage systemic administration.

Thus, virotherapy has proven to be safe in humans, but its efficacy needs further development. Towards this, different types of genetic modifications have been implemented and tested in animal models: first, simple backbone reengineering based on less attenuated genetic backgrounds. Second, changes resulting in targeted entry in cancer cells combined with other targeting types. And third, envelope exchange combined with cancer-cell targeting. Moving to clinical trials, recombinant viruses based on these three types of modification will require increasing levels of added safety precautions.

Several oncolytic viruses were engineered based on extremely attenuated genetic backbones. For example, MV-CEA and MV-NIS have been engineered on an infectious cDNA backbone [13], with mutations limiting the function of its anti-innate immunity proteins [5]. Since these mutations may limit viral replication in certain cancer cases, their correction may improve oncolytic efficacy. Indeed, there is a precedent for striking improvement of oncolytic efficacy by simple backbone reengineering: T-VEC, the herpes simplex virus 1–based vector approved as cancer therapeutic in the US and Europe, was generated based on transferring cancer specificity–conferring mutations from an over-attenuated laboratory strain to a wild-type backbone and arming the virus with a gene encoding GM-CSF [17].

Recombinant viruses with retargeted tropism have shown promising efficacy in cancer preclinical models, [10, 18] but none have yet advanced to clinical trials. This is due in part to the thorough and complex process regulating new clinical grade drugs, mandating availability of generous funding for their development and safety testing [7]. We also note that chimeric viruses may also represent a safety concern for the patient contacts that will not have preexisting neutralizing immunity. For MeV, this concern can be addressed by including in the attachment protein of chimeric viruses mutations that inactivate cell entry through the epithelial receptor nectin-4 and thus interfere with host exit and contagion [19]. For other viruses, different mechanisms for inactivation are being developed.

## Is immunovirotherapy the next frontier?

While producing and validating new recombinant viruses for clinical use is complex and slow, the combination of already approved oncolytic viruses with anticancer drugs is easier to implement. Immune checkpoint inhibitors are a particularly attractive new class of anticancer drugs. They were developed based on the observation that the immune system recognizes and is poised to eliminate cancer cells as well as virus-infected cells but is held in check by inhibitory receptors and their ligands [20]. Drugs interrupting these immune checkpoints can elicit antitumor immunity and mediate durable cancer regression. Can these checkpoint inhibitors be combined with replication-competent viruses to enhance their efficacy while limiting tissue damage of antiviral immune responses?

The answer is yes, provided that viral replication is restricted to cancer cells and that it precedes and primes the antitumor immune response. In its initial phase, oncolysis depends on efficient viral replication within tumor cells and induction of cell death. In its second phase, a potent innate immune reaction occurs at the site of infection and cell death, leading first to extensive immune-mediated tumor cell killing and then to the priming of adaptive T cell responses against the tumor antigen released by dying tumor cells [21]. Given the impressive efficacy of immune checkpoint inhibitors, it was sought to assess whether these would amplify oncolytic virus-induced tumor-specific immune responses. Indeed, it was shown that the combination of one checkpoint blockade (anti-CTLA-4) with a recombinant MeV enhances oncolytic therapy [22], as does the combination of another blockade (anti-PD-L1 antibody) with vesicular stomatitis virus [23], providing proof of principle. While at this point only encouraging preliminary results for metastatic malignant melanoma have been published [24], several clinical trials evaluating different combinations of oncolytic viruses and immune checkpoint inhibitors are ongoing, and many more are being planned.

To summarize, viruses are rapidly emerging as a promising new modality in the fight against cancer. Their dual mechanism of action, comprising direct intratumor spread/oncolysis followed by inflammatory boosting of the antitumor T cell response, makes them ideal partners for combination with immune checkpoint inhibitors and other immune-oncology agents. In addition, there is potential for synergy with conventional anticancer drugs. The opportunities for engineering, reshaping, and refinement of oncolytic viruses are vast: in the coming years, improved oncolytic viruses engineered for superior specificity, safety, potency, replicative fitness, ease of manufacturing, or other desirable characteristics will be developed. An anticipated future challenge will be how to decide when and whether a newer virus prototype is ready to enter the clinical development pipeline, where it must challenge and displace its better-established predecessor.
